# Sleep and Lifestyle Habits of Medical and Non-Medical Students during the COVID-19 Lockdown

**DOI:** 10.3390/bs13050407

**Published:** 2023-05-13

**Authors:** Ivana Pavlinac Dodig, Linda Lusic Kalcina, Sijana Demirovic, Renata Pecotic, Maja Valic, Zoran Dogas

**Affiliations:** Department of Neuroscience and Split Sleep Medicine Center, University of Split School of Medicine, 21000 Split, Croatia; ivana.pavlinac@mefst.hr (I.P.D.); linda.lusic@mefst.hr (L.L.K.); sijanademii@gmail.com (S.D.); renata.pecotic@mefst.hr (R.P.); maja.valic@mefst.hr (M.V.)

**Keywords:** lifestyle, sleep, mood, COVID-19, medical students

## Abstract

It has been shown that the measures of social distancing and lockdown might have had negative effects on the physical and mental health of the population. We aim to investigate the sleep and lifestyle habits as well as the mood of Croatian medical (MS) and non-medical students (NMS) during the COVID-19 lockdown. The cross-sectional study included 1163 students (21.6% male), whose lifestyle and sleep habits and mood before and during the lockdown were assessed with an online questionnaire. The shift towards later bedtimes was more pronounced among NMS (~65 min) compared to MS (~38 min), while the shift toward later wake-up times was similar in both MS (~111 min) and NMS (~112 min). All students reported more frequent difficulty in falling asleep, night-time awakenings and insomnia (*p* < 0.001) during lockdown. A higher proportion of MS reported being less tired and less anxious during lockdown compared to pre-lockdown (*p* < 0.001). Both student groups experienced unpleasant moods and were less content during lockdown compared to the pre-lockdown period (*p* < 0.001). Our results emphasize the need for the promotion of healthy habits in the youth population. However, the co-appearance of prolonged and delayed sleep times along with decreased tiredness and anxiety among MS during lockdown reveals their significant workload during pre-lockdown and that even subtle changes in their day schedule might contribute to the well-being of MS.

## 1. Introduction

Due to the outbreak of the novel coronavirus disease, COVID-19 (Coronavirus disease 2019), which spread considerably worldwide, the World Health Organization declared a pandemic. To contain and mitigate the devastating effects of pandemics, national governments as well as the national and local public health authorities of most European countries applied measures of social distancing and lockdown. These measures, including the restriction of social contacts and switching to online teaching modes in schools and universities, had convincing success in controlling the spread of the virus. Still, the long-term home confinement and possible negative effects of lockdown on the physical and mental health of the population are yet to be determined in the aftermath of the pandemic.

It has been shown that, during the COVID-19 pandemic, the lockdown restrictions and home confinement led to a reduction in physical activity and the adoption of sedentary behaviors, which had deleterious effects on neuromuscular, metabolic, and cardiovascular systems [1,2]. Furthermore, the restriction of social interactions and physical distancing might disrupt sleep quality and cause cognitive and psychological distress [3,4]. The dose-dependent manner of sleep disruption and perceived stress assumes that higher levels of stress lead to a more pronounced decline in sleep quality, which therefore, aggravates stress and its consequences [4]. This psychological burden might promote unhealthy changes in sleep and lifestyle habits, such as an increase in tobacco and alcohol consumption along with the decrease in physical activity during lockdown [5]. Having in mind that the severity and the novelty of large epidemics may induce a greater level of stress than the risk of infection or death itself [6], the COVID-19 outbreak might be considered as a severe stressful event, with significant consequences on sleep and lifestyle behaviors.

Similar negative effects of lockdown were also observed among the student population. Namely, during lockdown, students were less physically active, had more sleep problems and worse subjective sleep quality, as well as decreased self-perceived well-being [7,8]. Furthermore, a deterioration in the mental health of university students was noted in increased anxiety, depression, stress levels, and fear of COVID-19 [9,10,11]. Mental health problems were associated with both changes in lifestyle and sleep habits, as poor sleep habits resulted in decreased sleep quality, consequently leading to poor motivation, fatigue, and stress [12,13]. Along with the change in sleep habits, one of the notable changes among students’ lifestyle habits during the pandemic was the increased use of technology, such as mobile phones, online gaming, and social media [14,15]. It has been shown that the frequency of social media use correlated with the prevalence of mental health problems during lockdown, possibly due to exposure to profuse information on virus spread and people’s reaction on social media [16,17]. Thus, one might presume that changes in habits during lockdown resulted in a vicious cycle of unfavorable behaviors.

Given the delicacy of psychological changes during early adulthood and the pressure of high academic demands, it is not surprising that the student population is highly susceptible to sleep problems [18,19,20,21]. It has been shown that the prevalence of a specific constellation of personality traits, such as ambitiousness, perfectionism, competitiveness, obsessive-compulsive behaviors, and impatience, is more common among medical students (MS) [22]. The combination of these traits and a demanding and stressful environment during the medical training process might have unfavorable effects on psychological health [23,24,25]. Since stress itself is highly prevalent among MS and has potent adverse effects on sleep, it might be presumed that lockdown itself further impaired sleep, resulting in mood changes and mental health deterioration [18,19]. It has been shown that a low physical activity and high screen time, which are possible lockdown accompanying factors, increase the risks of mental health problems and poor sleep quality among college students [26]. One might presume that switching to an online teaching mode at universities could even further promote these negative lockdown-related behaviors. On the contrary, optimizing physical activity, nutrition, and sleep contributes to the well-being and better mental health of youths [24]. Finally, considering the importance of healthy sleep and lifestyle habits for mental and overall well-being in the student population, the changes in sleep and lifestyle habits in youths during lockdown are of specific interest.

Thus, this study was performed to investigate and compare changes in sleep and lifestyle habits, as well as the mood of Croatian medical and non-medical students during the COVID-19 lockdown.

## 2. Materials and Methods

The protocol of this study was approved by the Biomedical Research Ethics Committee at the University of Split School of Medicine. All procedures were carried out in accordance with the ethical standards from the 1964 Helsinki Declaration. All participants were presented with the informed consent electronic form in which the research protocol and activities were explained, along with the remainder that participation was voluntary and anonymous. Participants were also informed that they could stop participating at any time while filling in the questionnaire. Following the explanation of the study purpose and content, all participants agreed to participate by clicking a tick box.

### 2.1. Participants

A total of 1163 (21.6% males) Croatian students, of which 652 were MS and 511 were non-medical students (NMS), participated in this study. Non-medical students were studying Humanities and Social Sciences, Theology, Pedagogy, Law, Economics, Political science, Psychology, Science, Veterinary, Pharmacy, Dental medicine, Laboratory diagnostics, Chemical and environmental technology, Civil Engineering, Architecture and Geodesy, Polytechnics, Engineering, Electrical engineering and computing, Information technology, as well as Police and Art academies. The level of these non-medical studies was undergraduate (university and professional level), integrated undergraduate and graduate, or graduate (university and specialist level). The medical school program was an integrative undergraduate and graduate university study, with the curriculum governed by Bologna regulations, principles, and recommendations. The medical school program lasts six years and contains 5500 h of direct teaching, with classes organized in blocks for each subject and not divided into semesters.

### 2.2. Methods

During and immediately after the first COVID-19 lockdown in Croatia (1–15 May 2020) an online cross-sectional survey was conducted. The questionnaire was distributed through social media platforms and e-mail messages to students. The validated and reliable self-administered questionnaire consisted of five sections: Demographics, Teaching, Lifestyle, Sleep, and Mood changes [27]. The Demographics section assessed age, gender, weight, height, current living place, attended school or university, while Teaching during lockdown assessed changes in teaching mode and quality, motivation for learning, and stressfulness of the classes. The Lifestyle section investigated cigarette and marijuana smoking, consumption of coffee, alcohol and pharmacologically active substances, employment of physical activity, learning, watching TV, usage of social media and nights out prior to and during lockdown among participants. The Sleep section assessed bedtimes and wake-up times, sleep latency, night-time awakenings, daytime naps, sleep problems (insomnia and difficulty in falling asleep) prior to and during the lockdown. The feeling of being chronically tired and the presence of chronic diseases were also tested. The self-assessment of calmness, rest, contentment, anxiety, anger, fear, discouragement, and sadness, on a Likert scale from 1 to 4 (1—Not at all, 2—Somewhat, 3—Moderately, 4—Very much so) before and during lockdown, was performed in the Mood changes section.

### 2.3. Statistical Analysis

The statistical package SPSS was used for the statistical analysis. Mean with standard deviation or median with interquartile range was calculated following the assessment of the data distribution (Kolmogorov–Smirnov test). For data in categories, frequencies with percentages were reported and group differences were assessed following chi-squared, Fisher exact test, McNemar test for paired categorical comparisons, and Friedman test. Wilcoxon signed rank test or Student’s *t*-test for paired samples was performed for repeated measurement assessment, while the Mann–Whitney U test or Student’s *t*-test was performed for independent groups, depending on the distribution of data. Statistical significance was set at *p* < 0.05.

## 3. Results

The median age of the study participants was 22 (IQR 20–23), and the predominant gender among both MS and NMS was female (78.4% and 78.5%, respectively). The demographic data of the study participants are shown in Table 1. Most students followed the restrictions imposed by national and local public health authorities in full or most of the time during lockdown. The obedience rate was somewhat higher among MS compared to NMS (98% vs. 92.9%, respectively, *p* < 0.001), as shown in Table 1.

### 3.1. Teaching

Changes in the organization of teaching during the COVID-19 lockdown are summarized in Table 1. In the case of NMS, there were somewhat more online classes to the full extent (50.2% vs. 42.8%), but less preparation at home than in the case of MS (3.1% vs. 10.4%, respectively, *p* < 0.001). Most of the participants (72.5% MS and 69.7% NMS) considered regular classes before lockdown to be better than online classes during lockdown. The motivation for learning was higher during the pre-lockdown period (in 65.9% MS and in 66.2% NMS). Still, a higher motivation for learning during lockdown was observed to be higher among NMS in comparison to MS (10.9% vs. 6.8%, respectively, *p* = 0.02, Table 1). Regarding academic demands, similar proportions of MS were more stressed over regular classes before and online classes during lockdown (35.5% vs. 36.5%). However, the proportion of NMS who found online classes more stressful was higher than the proportion of those who found regular classes more stressful (58.8% vs. 17.0%, respectively). Furthermore, more MS than NMS considered regular classes before lockdown to be more stressful (35.5% vs. 17.0%, respectively), whereas more NMS than MS considered online classes during lockdown more stressful (58.8% vs. 36.5%, respectively, *p* < 0.001, Table 1).

### 3.2. Sleep Habits

**Figure 1:**
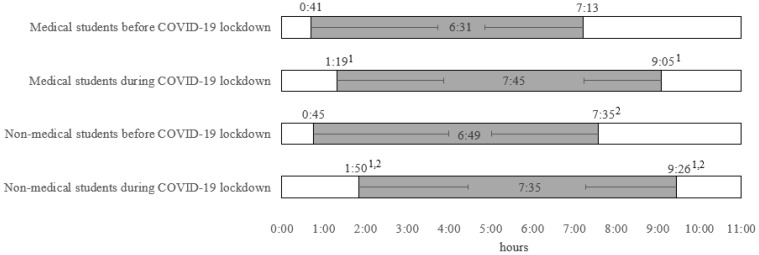
Sleep pattern changes during the COVID-19 lockdown. ^1^ Significantly different from the pre-lockdown period, *p* < 0.001. ^2^ Significantly different from medical students, *p* < 0.03. Student’s *t*-test was used for analysis.

NMS woke up later in comparison to MS during both the pre-lockdown (*p* = 0.023), and lockdown periods (*p* = 0.002), and went to sleep later than MS during lockdown (*p* < 0.001, Figure 1). A significant shift in bedtime and wake-up times was reported in both MS, and NMS, with later bedtimes and wake-up times in both groups of respondents (*p* < 0.001, Figure 1). The relative shift in bedtime was more pronounced among NMS (~65 min) compared to MS (~38 min), while the shift in wake-up times was similar in both MS (~111 min) and NMS (~112 min), as shown in Figure 1. The time needed to fall asleep, i.e., sleep latency, was longer in NMS in comparison to MS during both the pre-lockdown (20.6 ± 18.5 vs. 18.5 ± 16.4 min, respectively, *p* = 0.048) and lockdown periods (32.4 ± 29.8 vs. 28.3 ± 28.3 min, respectively, *p* = 0.020). Furthermore, sleep latency was prolonged in both respondents’ groups during lockdown (*p* < 0.001). An increase in self-reported insomnia, problems in falling asleep, and nighttime awakenings (*p* < 0.001) were noted in both respondents’ groups, whereas a decrease in tiredness was found only in MS during the lockdown period compared to pre-lockdown (*p* < 0.001, Table 2). MS reported, significantly and more frequently, to be chronically tired than NMS prior to lockdown (54.0% vs. 34.5%, *p* < 0.001, Table 2), whereas NMS reported more frequently being tired during lockdown (38.9% vs. 29.9%, *p* = 0.001). There was a significant reduction in the proportion of respondents feeling tired among the MS during lockdown compared with pre-lockdown (*p* < 0.001), but not among NMS (*p* = 0.16).

### 3.3. Lifestyle Habits during the Pre-Lockdown Period

During pre-lockdown, there were considerable differences in lifestyle habits among MS and NMS. Namely, a higher proportion of MS regularly exercised during pre-lockdown (67.3% vs. 56.2%, *p* < 0.001) and spent more time per day studying in comparison to NMS (174.7 ± 84.7 vs. 97.0 ± 70.8 min, *p* < 0.001). On the contrary, a higher proportion of NMS, compared to MS, smoked cigarettes (25.2% vs. 16.1%, *p* = 0.005, Table 1), or marijuana (8.2% vs. 4.9%, *p* = 0.022, Table 1), went out at night once or more per week (61.8% vs. 47.9%, *p* < 0.001), and spent more time per day watching TV (45.4 ± 45.1 vs. 37.9 ± 39.5 min, respectively, *p* = 0.002), or using computers (92.9 ± 78.3 vs. 61.7 ± 63.6 min, *p* < 0.001) during pre-lockdown. No differences in alcohol consumption, time spent on social networks, or using cell phones between medical and NMS during the pre-lockdown period were observed.

### 3.4. Lifestyle Habits during the Lockdown Period

The changes in lifestyle habits during lockdown in MS and NMS are summarized in Table 3. The alcohol consumption among both groups of students decreased during lockdown in comparison to pre-lockdown (*p* < 0.001, Table 3). During lockdown, there was an increased proportion of both groups of students who regularly exercise (*p* < 0.001, Table 3). However, the duration of physical activity during lockdown decreased in MS from 58.6 ± 26.9 min to 50.3 ± 26.7 (*p* < 0.001) minutes and, in NMS, from 59.0 ± 32.7 min to 48.0 ± 30.1 (*p* < 0.001) minutes compared to the pre-lockdown period (Table 3). The proportion of both MS and NMS who exercised four or more times per week increased during lockdown (from 26.4% to 58.4%, and from 30.3% to 52.8%, respectively, *p* < 0.001).

The use of cell phones, TV, social networks, and computers increased in both MS and NMS (*p* < 0.001, Table 3), while study time decreased only in MS during lockdown (*p* < 0.001, Table 3). A higher proportion of both MS and NMS believed that social networks and TV are distractors for sleep (*p* < 0.001, Table 3) and learning (*p* < 0.001, Table 3) during lockdown compared with the pre-lockdown period.

### 3.5. Changes in Mood during Lockdown

There was an increase in the rate of unpleasant moods of fear and discouragement during lockdown compared to pre-lockdown in both MS and NSM (*p* < 0.001, Table 4). MS were more frequently calm (*p* < 0.001), while both groups of students were more frequently rested and less frequently content during lockdown than during pre-lockdown (*p* < 0.001, Table 4).

Finally, there was a significant decrease in anxiety among MS (*p* < 0.001), whereas NMS were angrier (*p* < 0.001) and sadder (*p* < 0.001) during lockdown compared with the pre-lockdown period (Table 4).

## 4. Discussion

According to the results of the current study, a substantial change in various sleep and lifestyle habits was reported during the implemented measures associated with the COVID-19 pandemic in Croatia. We found that MS and NMS exhibited a comparable shift in wake-up times during the COVID-19 lockdown, whereas the shift in bedtime was more pronounced among NMS compared to MS. Furthermore, during lockdown, our subjects more frequently reported sleep problems, such as difficulties falling asleep, nighttime awakenings, and insomnia. Still, despite an increased incidence of reported sleep problems and prolonged sleep latency, MS were less tired during lockdown in comparison to the pre-lockdown period. Both MS and NMS were experiencing unpleasant moods (fear and discouragement) more frequently and were less content during lockdown compared to the pre-lockdown period. Both groups considered regular classes before lockdown to be better compared with online classes during lockdown, but MS found both types of classes similarly stressful. NMS, in a significantly greater proportion, considered online classes during lockdown more stressful. Still, there was a decrease in anxiety among MS during lockdown, which was not found in the NMS group.

It has been previously reported that the implementation of lockdown measures provoked shifts in sleep patterns, in terms of later bedtimes and wake-up times [28,29,30]. Indeed, the more pronounced shift in wake-up times than in bedtimes, as recognized in the current study, possibly indicates an accumulation of sleep debt during the pre-lockdown period in both respondents’ groups. Since a similar shift in wake-up times was observed in both MS and NMS and a shift in bedtimes was more pronounced in NMS, one might conclude that the sleep debt was more pronounced in MS than in NMS. Having in mind that medical students during the pre-lockdown period spent more hours per day studying than NMS, the more pronounced sleep debt observed in MS might be explained by the more demanding academic schedules and more stressful environment during medical education. Indeed, we believe that, due to the continuous burden of academic schedules, MS did not find the online teaching mode to be more stressful than the regular one, which was observed among NMS in our study. Our findings suggest that the online classes imposed by the pandemic were not well received among both student groups, expressing their overall preference for regular classes.

It is well known that altered circadian sleep patterns during adolescence appear due to a change in circadian phase preference and variability in melatonin secretion [31,32]. However, our findings show that previously the established shift of chronotypes toward eveningness [33] became even more pronounced during lockdown. Thus, it is likely that, following the suspension of ongoing university schedules and the consequent lack of strict schedule-related wake-up times in students, the evening phase preference became even more pronounced. Findings indicating that the shift toward eveningness peaks at 17 to 20 years of age [34,35,36] are in accordance with the evening preference found in the current study.

Our results imply that the absence of social schedules combined with the more common use of devices emitting artificial light modified the circadian clock, consequently even further delaying the bedtimes of students. We reported the more frequent use of computers, cell phones, and TVs during lockdown, which are known to have a dose-dependent relationship to delayed bedtimes and wake-up times [35]. Since the boundary between regular use of smartphones and addictive behavior has been recognized as being increasingly blurred [37], the more frequent use of cell phones reported in the current study should be afforded special consideration in the post-pandemic period, possibly having long-term consequences. All students also recognized social media as more frequently being a distractor of sleep and learning during lockdown. Given the association of daytime physical activity, eating behaviors, and light exposure with circadian rhythmicity [38], it is not surprising that the modification of these influences due to pandemic circumstances enabled students to sleep longer and closer to their natural circadian wake-up propensity rhythm. In fact, it has been proposed that the absence of socially constrained wake-up times enables one to catch up on the accumulated sleep debt [39]. Chronotypes have been shown as a relevant moderator of the investigated relationship, since sleep duration increases in evening-oriented participants, whereas it decreases in morning-oriented participants [40].

Social activities during nighttime are an additional exogenous factor contributing to delayed bedtimes and the dysregulation of sleep–wake-up patterns. However, in our study, there was a later bedtime even though nighttime social activities were less common in all students. Thus, one might presume that the urge to delay sleep times and wake-up times is a robust mechanism in the circadian physiology of youths.

It has been reported that physical isolation during the COVID-19 lockdown could have led to a decrease in sleep quality in students [3,7,18] and the general population [28]. More specifically, the disruption of the university schedule since the outbreak had detrimental effects on the sleep habits and sleep quality of students [3,4,7,28,41]. Our results support these findings in terms of more common sleep problems, such as difficulty in falling asleep, insomnia, and nighttime awakenings in both MS and NMS during lockdown. Still, one might presume that, despite the more prevalent sleep problems and prolonged sleep latency, decreased tiredness among MS might be the consequence of the prolongation of sleep time and shift in sleep–wake-up rhythm during lockdown. It should be underlined that it has been previously suggested that the impact of the pandemic on stress and sleep may not be entirely negative [42].

Home confinement and social isolation during the COVID-19 lockdown were associated with mood changes, increased levels of stress, and mental health and self-perceived well-being issues among the general population [5,43,44,45]. The negative mental health consequences of lockdown severely affected the student population in particular, which had an increased risk of even higher levels of depression, stress, and anxiety during the COVID-19 lockdown [44]. Students’ worries about the influence of lockdown on academic progression might have resulted in a decreased motivation for learning and the actual time spent studying during lockdown, as was reported in our subjects. Regarding moods, it is not surprising that both MS and NMS in our study reported an increase in unpleasant moods, such as fear and discouragement, and a decrease in contentment in comparison to the pre-lockdown period. However, only MS reported an increase in calmness and a decrease in anxiety. Whether these improvements in moods among MS are related to prolonged sleep and decreased tiredness, or a decrease in studying time per day [7], remain to be elucidated.

It has been shown that delayed sleep/wake-up cycles might have various adverse consequences on psychological well-being and academic performance [35]. Furthermore, a previous study indicated that both sleep quality and physical activity mediate the mental health consequences of a severe COVID-19 outbreak in the student population [3]. In terms of the physical activity of our sample, an increased proportion of students who exercised as well as increased frequency of exercise might have had protective effects on the mental health of students during lockdown. Still, having in mind that the duration of exercise shortened during lockdown in both student groups, the beneficial impact of exercise on mental health in our study remains questionable.

The online approach to investigate sleep and lifestyle changes during the COVID-19 lockdown might have introduced a few limitations to our results, specifically in terms of including generally more motivated students. Additionally, the research protocol did not include actigraphy or a sleep diary, which would provide objective information on the sleep and wake-up cycle. Still, as we used a validated questionnaire, we believe that it provided a reliable subjective assessment of the subjects’ sleep patterns. It should be emphasized that a high adherence to all or most restrictions during the COVID-19 lockdown provides confirmation that the investigated sample was, in fact, isolated during the pandemic. Furthermore, the higher proportion of females in our study fairly reflects the unequal gender distribution seen across medical studies in Croatia.

## 5. Conclusions

The current research established delayed sleep–wake-up pattern during the COVID-19 lockdown in Croatia, with a more prolonged sleep time among MS. Despite having a prolonged sleep latency and there being an increase in the frequency of sleep problems, such as insomnia, difficulty in falling asleep, and nighttime awakenings among students, the prolonged sleep time might have resulted in decreased tiredness and anxiety among MS, indicating their significant workload in pre-lockdown and that even subtle changes in their day schedule might contribute to the well-being of MS. It appears that MS benefited from a less structured schedule and a decreased academic burden in terms of decreased anxiety and tiredness. Taking into account the recommendations of the Sleep Research Society and National Institutes of Health, aimed at increasing the proportion of students with sufficient sleep duration [46], the results of our study further emphasize the importance of adoption of these recommendations, specifically during the COVID-19 lockdown. Hence, these findings should be of interest to both universities and policymakers in order to use an evidence-based approach in planning interventions alleviating the long-term impact of the COVID-19 pandemic on sleep and lifestyle habits. Therefore, our findings encourage an increased awareness of the relevance of sleep patterns and underline the need for constructive and evidence-based measures improving sleep quality during the COVID-19 pandemic in students. Furthermore, it should be taken into account that the changes in both sleep and lifestyle habits reported in this study may again emerge during unprecedented circumstances similar to the COVID-19 pandemic, disrupting well-established schedules in the student population.

## Figures and Tables

**Table 1:** Demographic and teaching data of the study respondents.

	Medical Students *n* = 652	Non-Medical Students *n* = 511	*p*
Gender, *n* (%)			
Male	141 (21.6)	110 (21.5)	0.97 ^1^
Female	511 (78.4)	401 (78.5)	
Age, median (IQR)	22 (20–24)	22 (20–23)	0.008 ^2^
Restriction obedience, *n* (%)			
Following all the restrictions	397 (60.9)	244 (49.8)	<0.001 ^3^
Follow restrictions most of the time	242 (37.1)	211 (43.1)	
Follow restrictions occasionally	11 (1.7)	32 (6.5)	
Do not follow restrictions	2 (0.3)	3 (0.6)	
Cigarettes smoking (YES), *n* (%)	105 (16.1)	129 (25.2)	0.005 ^1^
Marijuana smoking (YES), *n* (%)	32 (4.9)	42 (8.2)	0.022 ^1^
Teaching			
Classes organization, *n* (%)			
Online classes to full extent	279 (42.8)	246 (50.2)	<0.001 ^3^
Online classes to a shortened extent	300 (46)	222 (45.3)	
Self-preparation of teaching materials from home	68 (10.4)	15 (3.1)	
Classes are postponed	1 (0.2)	2 (0.4)	
Other	4 (0.6)	5 (1)	
Classes quality, *n* (%)			
Regular classes BEFORE lockdown were better	473 (72.5)	340 (69.7)	0.11 ^1^
Online classes DURING lockdown are better	60 (9.2)	36 (7.4)	
The quality is equal	119 (18.3)	112 (23.0)	
More stressful classes, *n* (%)			
Regular classes BEFORE lockdown	231 (35.5)	83(17.0)	<0.001 ^1^
Online classes DURING lockdown	237 (36.5)	287 (58.8)	
Both	150 (23.1)	91 (18.6)	
Classes are never stressful/tiring	32 (4.9)	27 (5.5)	
Motivation for learning, *n* (%)			
Higher BEFORE lockdown	429 (65.9)	323 (66.2)	0.02 ^1^
Higher DURING lockdown	44 (6.8)	53 (10.9)	
Equal before and during lockdown	178 (27.3)	112 (23)	

^1^ χ^2^ test; ^2^ Mann–Whitney U test; ^3^ Fisher’s exact test.

**Table 2:** Sleep complaints of medical and non-medical students prior to and during lockdown in medical and non-medical students.

	Medical Students			Non-Medical Students		
	Before Lockdown	During Lockdown	*p* ^1^	Before Lockdown	During Lockdown	*p* ^1^
Insomnia	106 (16.3)	151 (23.2)	<0.001	77 (15.7)	139 (28.4)	<0.001
Difficulty in falling asleep	144 (22.1)	252 (38.7)	<0.001	109 (22.2)	207 (42.2)	<0.001
Nighttime awakening	123 (18.9)	203 (31.1)	<0.001	106 (21.6)	163 (33.3)	<0.001
Tiredness	352 (54.0)	195 (29.9)	<0.001	178 (34.8)	199 (38.9)	0.16

All data are shown as frequency (percentage). ^1^ McNemar test.

**Table 3:** Lifestyle habits of medical and non-medical students prior to and during lockdown in medical and non-medical students.

	Medical Students			Non-Medical Students		
	Before Lockdown	During Lockdown	*p*	Before Lockdown	During Lockdown	*p*
Exercise						
Incidence, *n* (%)	439 (67.3)	512 (78.5)	<0.001 ^1^	290 (56.8)	337 (65.9)	<0.001 ^1^
Average duration (min), mean ± SD	58.6 ± 26.9	50.3 ± 26.7	<0.001 ^2^	59.0 ± 32.7	48.0 ± 30.1	<0.001 ^2^
Frequency of exercise, *n* (%)						
Every day	28 (6.4)	139 (27.1)	<0.001 ^3^	30 (10.3)	92 (27.3)	<0.001 ^3^
4–5 times/week	88 (20.0)	160 (31.3)		58 (20.0)	86 (25.5)	
2–3 times/week	187 (42.6)	101 (19.7)		110 (37.9)	68 (20.2)	
1–2 times/week	102 (23.2)	91 (17.8)		69 (23.8)	72 (21.4)	
1–2 times/month	34 (7.7)	21 (4.1)		23 (7.9)	19 (5.6)	
Alcohol consumption, *n* (%)						
Never	157 (24.1)	385 (59.0)	<0.001 ^4^	181 (35.4)	335 (65.6)	<0.001 ^4^
1 drink/month	237 (36.3)	124 (19.0)		154 (30.1)	84 (16.4)	
Up to 3 drinks/week	149 (22.9)	97 (14.9)		78 (15.3)	56 (11.0)	
Up to 7 drinks/week	39 (6.0)	24 (3.7)		35 (6.8)	11 (2.2)	
Up to 15 drinks/week	10 (1.5)	3 (0.5)		12 (2.3)	6 (1.2)	
More than 15 drinks/week	1 (0.2)	0 (0.0)		5 (1.0)	3 (0.6)	
Other	59 (9.0)	19 (2.9)		46 (9.0)	16 (3.1)	
Minutes per day spent…, mean ± SD						
Studying	174.7 ± 84.7	145.7 ± 89.9	<0.001 ^2^	97.0 ± 70.8	94.4 ± 79.8	0.40 ^2^
Watching TV	37.9 ± 39.5	77.8 ± 61.3	<0.001 ^2^	45.4 ± 45.1	80.4 ± 65.9	<0.001 ^2^
On social networks	117.1 ± 66.1	141.6 ± 76.4	<0.001 ^2^	124.2 ± 69.9	139.8 ± 75.5	<0.001 ^2^
On computer	61.7 ± 63.6	117.6 ± 82.5	<0.001 ^2^	92.9 ± 78.3	147.3 ± 89.1	<0.001 ^2^
Using cell phone	35.0 ± 39.0	51.9 ± 53.6	<0.001 ^2^	34.1 ± 37.2	51.4 ± 54.9	<0.001 ^2^
Social network and TV disturbing…, *n* (%)						
Sleep	318 (49)	403 (62)	<0.001 ^1^	242 (49)	287 (59)	<0.001 ^1^
Learning	351 (54)	451 (69)	<0.001 ^1^	279 (57)	320 (65)	<0.001 ^1^

All data are shown as mean ± SD or frequency (percentage). ^1^ McNemar test; ^2^ Student’s *t*-test; ^3^ Wilcoxon signed rank test; ^4^ Friedman test.

**Table 4:** Mood changes in medical and non-medical students prior to and during lockdown.

	Medical Students			Non-Medical Students		
	Before Lockdown	During Lockdown	*p* ^1^	Before Lockdown	During Lockdown	*p* ^1^
Calmness	2.54 ± 0.8	2.77 ± 0.8	<0.001	2.73 ± 0.7	2.66 ± 0.9	0.085
Restfulness	2.08 ± 0.8	2.83 ± 0.9	<0.001	2.34 ± 0.7	2.70 ± 0.9	<0.001
Contentment	2.67 ± 0.7	2.49 ± 0.8	<0.001	2.76 ± 0.7	2.42 ± 0.9	<0.001
Anxiety	2.44 ± 0.7	2.29 ± 0.8	<0.001	2.26 ± 0.7	2.34 ± 0.8	0.063
Anger	1.98 ± 0.7	2.03 ± 0.8	0.257	1.90 ± 0.7	2.12 ± 0.8	<0.001
Fear	1.63 ± 0.8	1.81 ± 0.9	<0.001	1.46 ± 0.7	1.75 ± 0.9	<0.001
Discouragement	1.95 ± 0.9	2.26 ± 0.9	<0.001	1.96 ± 0.9	2.33 ± 1.0	<0.001
Sadness	1.98 ± 0.7	2.04 ± 0.8	0.158	1.95 ± 0.7	2.12 ± 0.9	<0.001

All data are shown as mean ± SD. ^1^ Wilcoxon signed rank test.

## Data Availability

The datasets used and/or analyzed during the current study are available from the corresponding author upon reasonable request.
